# Impact of Coconut Oil and Its Bioactive Metabolites in Alzheimer’s Disease and Dementia: A Systematic Review and Meta-Analysis

**DOI:** 10.3390/diseases12110272

**Published:** 2024-11-01

**Authors:** Duaa Bafail, Abrar Bafail, Norah Alshehri, Noura Hamdi Alhalees, Ahmad Bajarwan

**Affiliations:** 1 Department of Clinical Pharmacology, Faculty of Medicine, King Abdulaziz University, Jeddah 22254, Saudi Arabia; 2 Nuclear Medicine Department, Le Centre Hospitalier Régional Universitaire, 37000 De tours, France; abrar.a.b@hotmail.com; 3 Pharmaceutical Care Department, East Jeddah General Hospital, Ministry of Health, Jeddah 22253, Saudi Arabia; nalshehri852@gmail.com; 4 Drug Information Department, Ministry of Health King Abdullah Medical Complex, Jeddah 23816, Saudi Arabia; dr.nona.alhalees@gmail.com; 5 Engineering Management, Old Dominion University, Norfolk, VA 23529, USA; aabajarwan@hotmail.com

**Keywords:** Alzheimer’s disease, coconut oil, cognition, dementia, meta-analysis

## Abstract

**Background/Objectives**: Alzheimer’s disease (AD) is the most common form of dementia and affects approximately 50 million individuals worldwide. Interest in coconut oil (CO) as a potential dietary intervention has surged owing to its substantial medium-chain triglyceride (MCT) content. Therefore, sustaining cognitive function and potentially slowing the progression of AD are crucial. This systematic review and meta-analysis evaluated the effects of CO and its bioactive metabolites on AD and dementia. **Methods**: The review protocol is registered in PROSPERO (CRD42023450435). Relevant research articles published between January 2015 and June 2023 were systematically searched. Seven studies met the predetermined eligibility criteria. Thematic analysis was utilized to synthesis the data about the qualitative features, while meta-analysis was employed for the quantitative findings. A meta-analysis was conducted to assess the standardized mean difference (SMD) and the corresponding 95% confidence interval (CI). Forest plots were generated using Review Manager 5.3 (RevMan 5.3). **Results**: The analysis revealed that all studies showed consistent results regarding the effects of CO on cognitive scores, with little variability in the true effects of CO on cognitive scores across the studies included in the meta-analysis. **Conclusions**: CO improved cognitive scores in patients with AD compared with those in the control group (*p* < 0.05). The results of this study add to the increasing amount of evidence indicating that MCTs found in CO might be a way to improve abilities and potentially slow the advancement of AD. The findings of this study may encourage the development of targeted dietary strategies and interventions for individuals at risk of or diagnosed with AD.

## 1. Introduction

Alzheimer’s disease (AD), a progressive neurological disorder, is the most common form of dementia and accounts for 60–80% of all dementia cases [1]. The estimated number of patients with dementia worldwide exceeds 50 million [2]. Between 2015 and 2018, the pooled prevalence rate of dementia in Mainland China was reported to be 7.4% [3]. Dementia is associated with functional and structural alterations in the brain, aligning with the anomalies identified in individuals with moderate cognitive impairment and AD [4]. This condition is characterized by intraneuronal deposits of neurofibrillary tangles and extracellular amyloid-β plaques. Current medications alleviate symptoms without affecting disease progression [5]. Given the high frequency of AD, the growing burden of dementia, and the lack of progress in discovering effective AD treatments, the search for novel drugs is of substantial importance. Coconut oil (CO) has a long history of ethnopharmacological use among Indians and has recently gained prominence as a potential neuroprotective functional food. This is largely due to the well-established link between oxidative stress and neurodegeneration as well as the antioxidative properties of CO. The fatty acid profiles of the two primary forms of CO, copra oil and virgin CO (VCO), are comparable; however, the latter contains more nutrients (e.g., vitamin E) and dietary bioactive substances (e.g., polyphenols) [6]. The unique chemical composition of CO, which is rich in medium-chain fatty acids (MCFAs) such as caprylic acid (C-8:0) (8%), capric acid (C-10:0) (7%), lauric acid (C-12:0) (49%), myristic acid (C-14:0) (18%), palmitic acid (C-16:0) (8%), stearic acid (C-18:0) (2%), oleic acid (C-18:1) (6%), and linoleic acid (C-18:2) (2%) [4,7], has prompted researchers to investigate its nutritional and therapeutic effects. These MCFAs are primarily responsible for the unique positive effects of VCO. It has been reported that CO enhances brain function and promotes cardiac health; it has also been reported to possess antioxidant [8], anti-inflammatory [9], hypolipidemic, and antithrombotic properties [10]. However, there is no conclusive literature on the efficacy of CO for the treatment of dementia in AD [11]. Due to their high concentration of medium-chain fatty acids (MCFAs) and bioactive metabolites, which include antioxidants and anti-inflammatory chemicals, coconut oil preparations, especially virgin coconut oil (VCO) and copra oil, are recognized for their potential therapeutic benefits. While copra oil is recovered from dried coconut meat, VCO is obtained by cold pressing, which retains more nutrients and bioactive substances. These preparations are made from the flesh of the coconut using various methods. Medium-chain triglycerides and other bioactive substances found in CO have been linked to the potential advantages of oil for the neurological system, as they have been reported to act as brain fuel [9]. The liver absorbs and processes MCTs, metabolizing them into ketones that may act as substitutes for glucose in brain cells, potentially reducing the progression of various disorders [4,7]. Ketone bodies (KBs), produced in the liver during fat metabolism, serve as an alternative energy source to glucose, particularly during periods of fasting or carbohydrate restriction. KBs, such as β-hydroxybutyrate, have been shown to improve cognitive function by enhancing mitochondrial function and reducing oxidative stress. This metabolic shift offers neuroprotective benefits and has the potential to slow the progression of neurodegenerative diseases [1,3,8]. This systematic review and meta-analysis assessed the effects of CO on AD and dementia based on cognitive scores. In particular, this study highlights the role of coconut oil as a source of medium-chain triglycerides (MCTs), which may promote the production of ketone bodies, providing an alternative energy source for brain cells. Additionally, we discuss the importance of incorporating other brain-healthy foods, such as omega-3 fatty acids, antioxidants, and vitamins, to create a comprehensive dietary approach aimed at enhancing cognitive function and overall brain health.

## 2. Materials and Methods

The Preferred Reporting Items for Systematic Reviews and Meta-Analyses (PRISMA) standards were used in the current study [12].

### 2.1. Search Strategy

A comprehensive search of relevant scientific literature was using the following databases: PubMed (Medline), ScienceDirect, Scopus, Google Scholar, and Cochrane Central Register of Controlled Trials (CENTRAL). To identify studies on the effect of CO and its main bioactive compounds on AD and dementia, we considered the following key concepts and search terms. For AD and dementia, we used the following terms: “Alzheimer”, “dementia”, “cognitive impair”, “neurocognitive disorder”, “memory loss”, “cognitive decline”, and “senile dementia”. For coconut oil and its bioactive components, we included the terms “coconut oil”, “MCT oil”, “medium chain triglycerides”, “lauric acid”, “caprylic acid”, and “capric acid” to encompass relevant interventions and compounds. To combine these concepts effectively, we used Boolean operators (AND, OR) in our search strategy. Additionally, we used asterisks as wildcards to account for variations in word endings and improve search sensitivity. The complete search string for PubMed is presented in Supplementary File S1 (Table S1).

### 2.2. Eligibility Criteria

This systematic review and meta-analysis included studies published between January 2015 and July 2023 that investigated the effects of CO on human AD. Only original study designs were taken into consideration, such as qualitative and quantitative studies for content analysis and cohort, case–control, or randomized controlled trials (RCTs) for meta-analyses. Articles published in peer-reviewed journals were included, whereas review articles, editorials, meta-analyses, conference abstracts, and studies on nonhuman subjects were excluded. Additionally, studies lacking information on the effect of CO on AD or those with insufficient data and unclear methodologies were excluded.

### 2.3. Article Screening

A two-phase screening procedure identified the publications that will be analyzed. First, paper titles and abstracts were checked against the qualifying requirements. Two authors (D.B. and N.F.A.) conducted individual screenings of articles based on titles and abstracts to exclude irrelevant studies. Subsequently, a thorough full-text screening of the remaining studies was performed, and studies that met the eligibility criteria were finalized. Disputes regarding study selection were resolved by a third researcher (A.B.). We also checked the full-text reference lists to make sure no publications that could have been relevant had been overlooked. One reviewer (D.B.) was in charge of settling disputes at this second stage, and the reasons for elimination were documented. The selection process is visually represented in the PRISMA flowchart.

### 2.4. Quality Assessment

The quality of RCTs was assessed using the Cochrane Risk of Bias 2.0 (RoB 2.0) tool [13]. RoB 2.0 encompasses evaluation across five key domains: (1) bias arising from the randomization process, (2) bias due to deviations from the intended interventions, (3) bias due to missing outcome data, (4) bias in the measurement of the outcome, and (5) bias in the selection of reported results. The RoB 2.0 tool was visualized using the ROBVIS tool [14]. For non-randomized studies, the Newcastle–Ottawa scale was used. Only moderate- and good-quality studies were included in this systematic review and meta-analysis.

### 2.5. Data Extraction

**Figure 1:**
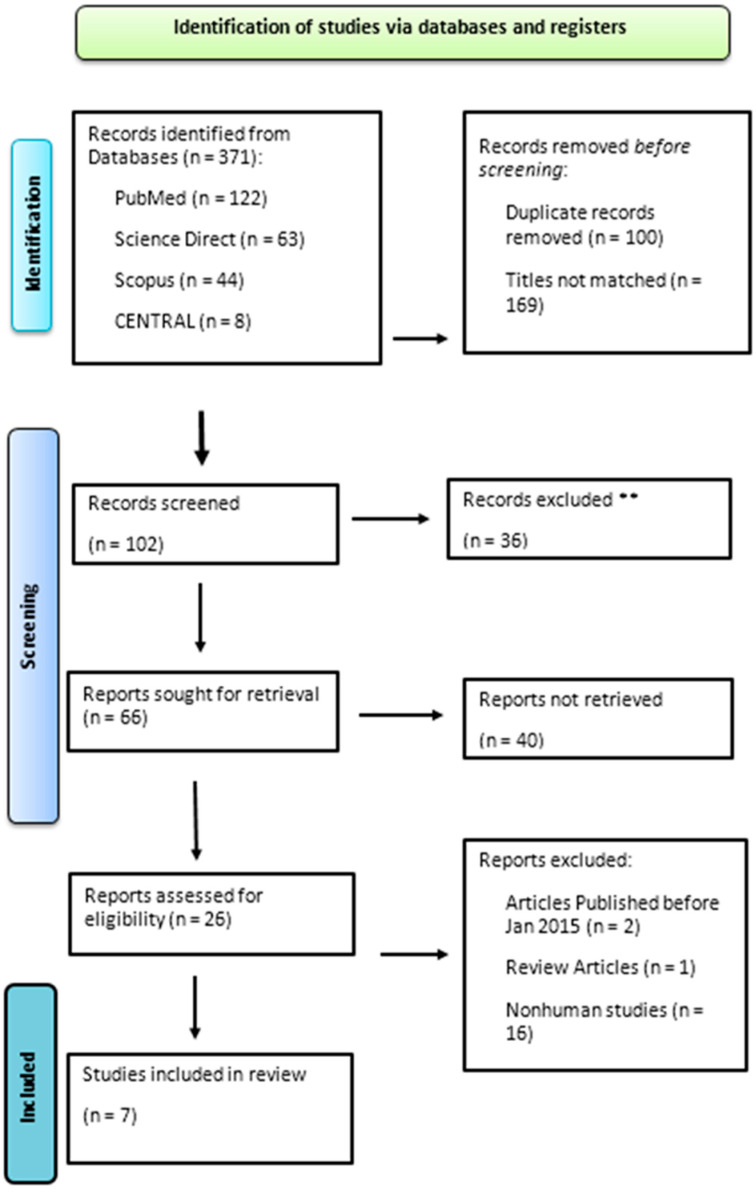
PRISMA flowchart presenting the exclusion and inclusion criteria for the review. ** exclusion based on unwanted outcome.

For data extraction, two reviewers (N.H.A. and D.B.) inserted information from the selected studies into an Excel spreadsheet using a pre-piloted extraction form. Disparities in the extracted data were addressed by a third reviewer (A.B.). In addition to searching databases, we manually examined the reference lists of the selected articles. We also reviewed citations from the final list of selected articles to determine whether they met our inclusion criteria. Studies in languages other than English were translated using Google Translate during the full-text screening in Figure 1. The study protocol was registered in the International Prospective Register of Systematic Reviews (PROSPERO) under registration number CRD42023450435.

### 2.6. Data Analysis

Importing the papers into NVivo 12 Pro, one reviewer (A.B.) synthesized the data using a thematic analysis. To do this, codes were applied to specific textual passages that represented aspects of the study and intervention as well as outcomes related to the primary objectives. Two reviewers confirmed the thematic analysis findings (N.H.A. and D.B).

Data from the included studies were synthesized and analyzed using a meta-analysis. We used the standardized mean differences (SMDs) of the two cognitive test scores, namely the mini-mental state examination (MMSE) and the seven-minute screen (7MS). The SMD was calculated as the difference in means between the CO intervention and control groups divided by the pooled standard deviation. A random-effects model was used to estimate the overall effect size, and the results were presented as forest plots.

Statistical analysis of the retrieved articles was performed using Review Manager (RevMan, Version 5.3; The Cochrane Collaboration, Copenhagen, Denmark). For each group or subgroup analysis, the chi^2^ test and I^2^ statistic were computed to assess and quantify the heterogeneity. A chi^2^ value of less than 0.05 was considered significant in indicating the presence of heterogeneity. The Cochrane Handbook for Systematic Reviews of Interventions was used to interpret the I^2^ values [15]. Specifically, I^2^ values were interpreted as follows: low heterogeneity (I^2^ < 25%), indicating minimal inconsistency among studies; moderate heterogeneity (I^2^ = 25–50%), suggesting moderate variability between studies; substantial heterogeneity (I^2^ = 50–75%), indicating significant heterogeneity requiring careful consideration; and considerable heterogeneity (I^2^ > 75%), signifying substantial diversity among studies. This approach allowed a comprehensive assessment of heterogeneity and informed subsequent analyses and interpretations in our systematic review and meta-analysis.

## 3. Results

**Figure 2:**
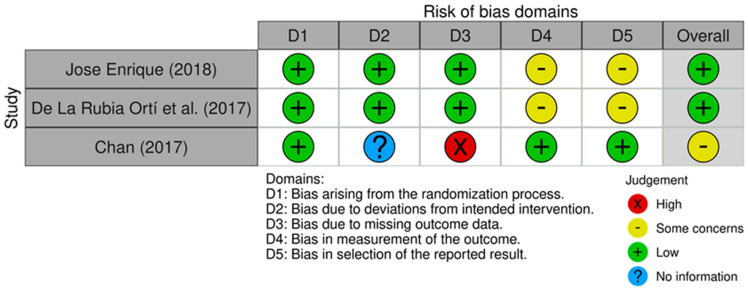
Risk of bias assessment of randomized controlled trials (RCTs) [16,17,18] using the ROB 2.0 tool and visualized by the ROBVIS tool. Two of the four studies were from the same author, so the identifier used for the study by de la Rubia Ortí et al. (2018) [18] was the first name of the first author, i.e., Jose Enrique (2018).

The initial literature search yielded a total of 371 studies. Duplicate experiments conducted (*n* = 100) were identified and removed. In total, 169 studies were excluded because their titles did not match the topics of interest. The remaining studies were screened based on the titles and abstracts, yielding 66 studies. The full texts of 26 studies were obtained. Following the retrieval of the full text, a rigorous assessment was conducted against the predetermined inclusion criteria. This in-depth evaluation resulted in the exclusion of 19 articles, leaving a final selection of 4 studies that provided a well-defined investigation of AD. Only seven studies that fulfilled the inclusion criteria in their entirety and were of moderate or high quality were considered for inclusion [16,17,18,19,20,21,22]. The results of the quality assessment are presented in Table 1 and Figure 2. Table 1 displays the methodological quality scores of qualitative studies. The range of total quality ratings was 2 to 10, meaning that no publication fully satisfied all the requirements for their research design. (An asterisk indicates that the study met the corresponding quality assessment criteria. A zero (0) denotes that the study did not meet the criteria for that specific quality indicator.)

Three studies [16,17,18] were RCTs, one [21] was a case–control study, and three were qualitative studies [19,20,22]. Three studies were conducted in Spain [17,18,21], while single studies were conducted in Malaysia [16], USA [19], and Sri Lanka [20], and Canada [22] are presented in Table 2.

### 3.1. Meta-Analysis

A total of four studies [16,18,21] were identified based on the eligibility criteria. The goal was to use various cognitive assessment scores to compare the improvements in memory and cognition in patients with AD. Three of the four studies mentioned cognition scores. Of the three remaining studies, one study did not provide comprehensive data for pooling results in the final table, but rather stated a significant improvement in pre- and post-test clock drawing test (CDT) scores in the control group (mean CDT −0.78571, 95% CI CDT: −1.50824 −0.06319; *p* = 0.035) [16].

Two studies used comprehensive cognitive assessment scales. One study [21] used the Mini-Examen Cognoscitivo (MEC) scale, which is a Spanish translation of the MMSE, while the other [18] evaluated individuals using the 7MS test. This test uses four individual measures: Benton’s test for temporal awareness, the CDT for visuospatial memory, a categorical verbal fluency test, and a free and cued selective reminder test for episodic memory. Owing to the lack of sufficient studies with pooled results, the approach adopted for statistical analysis involved evaluating the results of each of these studies, comparing the percentage improvement in results, and reaching a conclusion about their statistical significance using a paired-sample *T*-test.

To derive meaningful results from the two studies, we used the SMD of the two cognitive test scores, namely, the MMSE and 7MS. The total MMSE score was calculated out of 30, with the standard deviation provided for the demographics within the study, allowing for a comparison between the intervention and control groups.

The 7MS is a combination of four cognitive test scores, as previously mentioned. The total score for the 7MS is calculated in the study [18] out of 156, with a higher score indicating better performance. The study did not provide the scores for the entire sample, but rather provided individual test scores for the four tests, along with the standard error of the mean (SEM) for each observation. Therefore, the individual test scores and the SEM were converted into 7MS scores in total, along with the standard deviations calculated from the SEM for each observation.

**Figure 3:**
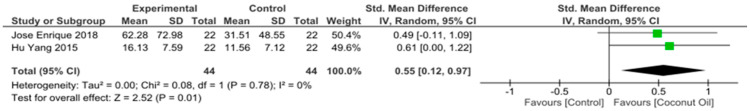
Forest plot of the studies by Jose Enrique (2018) and Hu Yang (2015) [21]. Two of the four studies were from the same author, so the identifier used for the study by de la Rubia Ortí et al. (2018) [18] was the first name of the first author, i.e., Jose Enrique (2018).

A forest plot comparing the intervention and control group scores on the MMSE and 7MS was created using the inverse variance method of statistical analysis with a random effects model. CIs were set at 95%. The mean scores for the intervention and control samples were added along with the standard deviations, and the SMD, also known as Cohen’s d for effect size, was used to compare the two scores. SMD was used because different scoring systems were used in the two studies. By comparing the standardized values, we were able to draw meaningful conclusions from the combination of these two studies. The forest plot is shown in Figure 3.

Heterogeneity tests of the included studies yielded a Tau^2^ of 0.00, indicating little variability in the true effects of CO on cognitive scores across the studies included in the meta-analysis. The chi-square test result was not statistically significant (Chi^2^ = 0.08, df = 1, *p* = 0.78), suggesting no significant heterogeneity among the studies’ effect sizes. The I^2^ value was 0%, indicating no heterogeneity among studies. Thus, all studies have shown consistent results regarding the effects of CO on cognitive scores. The overall effect of CO on AD was found to be statistically significant (Z = 2.52, *p* = 0.01), implying that CO had a positive impact on cognitive scores compared with the control group.

Both studies enrolled 44 patients and consequently had similar weights for the final score. The pooled standardized mean difference was 0.55 (95% CI, 0.12–0.97), which showed a medium effect size (according to Cohen’s d interpretation for effect size). With a Cohen’s d of 0.55, 70.9% of the patients in the intervention group had a score greater than the mean of those in the control group (Cohen’s U3), and the probability that a person picked at random from the intervention group consuming the CO product/CO-rich meal would have a higher score than a person picked at random from the control group (probability of superiority) was 65.1%. Based on the analysis of previous databases and studies, it is estimated that, on average, treating 5.4 individuals in the intervention group is necessary to achieve one additional positive outcome compared to the control group (https://rpsychologist.com/cohend/ (accessed on 13 August 2023)).

#### 3.1.1. MMSE Score

The maximum MMSE score was 30. A score of 25 or higher was considered normal. If the score was <24, the result was considered abnormal, indicating a possible cognitive impairment. For the MMSE scores reported by Hu Yang [18], a 38.92% improvement was observed in the population taking the CO product compared to the placebo, which showed minimal improvement. A paired-sample t-test was conducted to determine the effects of the coconut product. A significant difference between the MMSE score before the intervention (M = 11.61; SD = 6.85) and the MMSE score after the intervention (M = 16.13; SD = 7.59) [t (21) = 3.009, *p* < 0.05] was observed. Similarly, improvements were seen when the averages of the MMSE scores were compared on the basis of sex, with women showing slightly greater improvement in scores after receiving the treatment (39.70%) than men (36.99%). However, the difference in the mean scores between men and women was not statistically significant. (t = 1.3192, df = 20, *p* = 0.202). This is shown in Table 3.

#### 3.1.2. CDT Scores for Visuospatial Memory

Mean scores for the CDT were reported as part of the 7MS in a study by Jose Enrique [17]. The patients scored a maximum of 7 points, and a higher score was associated with better cognition and visuospatial memory. We analyzed the improvements in the performance of those with moderate disease in a descriptive manner while incorporating the entire sample size (i.e., moderate and severe disease patients) into the forest plot analysis. In contrast, CO-based product administration seemed to worsen the CDT scores in females. This observation showed a 50% decrease in the mean CDT scores of the women before and after the intervention.

#### 3.1.3. Benton’s Temporal Awareness Test

The scoring for this test is performed on the basis of errors from accurate judgment of time, wherein a score of 0 means the most errors made for the scoring system adopted in the study by de la Rubia Ortí et al. [18], and the maximum score is 113. A higher score indicates a good prognosis. As shown in Table 4, the intervention provided a greater benefit than the control, with a 142% score improvement after the intervention. The improvements in the control group were not substantial. Similarly, in the female population, the intervention group showed an improvement of 37.4% over the pre-test scores, whereas the control group exhibited a decrease in scores (34.9%).

#### 3.1.4. Verbal Fluency Test

The verbal fluency test assessed the patient’s capacity for fluent speech and semantic memory, with maximum scores of 20 points and 1 point, respectively, for each word spoken by the patient. Among males, there was no observable improvement in scores before and after the intervention, whereas the control group showed a slight improvement in post-test scores. Among female patients, the intervention group showed an improvement compared to the pre-test scores (33.3% improvement), whereas the control group experienced a decline in performance. A comparison of the post-test mean scores between the intervention and control groups was performed using an independent t-test. However, the results showed a statistically insignificant difference in means (t = 0.7793, df = 8, *p* = 0.45).

#### 3.1.5. Episodic Memory Test

An episodic memory test was performed using the free and cued selective reminder test, with a maximum score of 16. The better the patient’s recall of the figures presented to him or her, the better his or her score. In the male demographics, the scores showed an improvement in both the intervention and control groups; notably, a greater improvement was seen in the control group (150% increase in scores) than in the intervention group (41.3%). However, there was only one patient in the control group, which reduced the reliability of this observation. The study reported a statistically significant difference in the pre- and post-test scores; however, the only *p*-value provided was *p* < 0.01. The scoring of women seemed to continue the trend from other tests, where the women receiving the coconut product showed an improvement in their test scores (21.2%), whereas those in the control group showed a decline in their performance. This observation, however, when verified by an independent-sample *T*-test, did not prove a statistically significant difference between the mean values (t = 0.5446, df = 8, *p* = 0.600).

The enhanced test scores observed in males after receiving the placebo, particularly in Benton’s test, the mean verbal fluency test, and the mean episodic memory test, could be attributed to the placebo effect, where participants may experience perceived improvements due to their belief in receiving treatment. Additionally, baseline differences in cognitive function between groups or psychological factors, such as increased motivation or attention, might have influenced the results.

### 3.2. Qualitative Analysis

#### 3.2.1. MCTs

Medium-chain triglycerides (MCTs), which include medium-chain fatty acids (MCFAs), make up CO, which is not a single molecule. These molecules are usually present in significant concentrations in CO; around 65% of the components are MCTs, and 100% of the constituents are MCFAs. The metabolism of glucose, the brain’s main energy source, is essential for normal operation [19]. Crude MCTs have been proposed as a potential alternative energy source to enhance cognitive performance by breaking down KBs, even in the presence of glucose [20]. But the degree of this improvement seems to differ for a lot of people, and it might not work for everyone who receives treatment [22]. The efficacy of MCT as a therapy depends on the degree and course of AD. Without further processing, MCTs usually do not function as a store of energy because their fatty acids are unable to efficiently cross the blood–brain barrier (BBB) [20]. Fatty acids by themselves cannot appreciably influence β-amyloid plaques and neurofibrillary tangles due to their low BBB penetration. Therefore, it is more likely that the use of CO as an AD-related treatment will be associated with the byproducts of MCFA metabolism, such as KBs [19].

#### 3.2.2. Ketone Bodies

One process that has been studied in CO supplementation is the switch from glucose consumption to KB utilization, which provides brain tissues with an alternative energy source. For instance, acetoacetate (AcAc) and β-hydroxybutyrate (β-HB) are the two primary ketone bodies produced during ketone metabolism [20]. These ketone bodies, which can be derived from medium-chain triglycerides (MCTs) present in coconut oil (CO), enter neurons through distinct plasma membrane transporters compared to those utilized by glucose. Once inside the cells, they can be converted into ATP via the Krebs cycle [22]. Glucose hypometabolism in AD patients might result in a 20–25% glucose shortage. Increased ketone intake can make up for this deficiency, and there is no discernible difference in ketone uptake between AD and age- and health-matched controls [20].

#### 3.2.3. CO Effects with Specific Components of Metabolites

It seems that CO inhibits the release of Aβ peptides. Aβ peptides were shown to be significantly reduced by 0.1% CO. By decreasing ARF1 expression at the protein and mRNA levels, this inhibitory impact changed the expression of APP and the generation of Aβ peptides [19]. The secretory route that moves APP to the cell surface depends on the transport protein ARF1. The secretion of Aβ peptides is determined by the surface expression of APP [20]. CO has detrimental effects in addition to its potential to positively alter AD-related molecular pathways through KBs. While it is known that KBs can lower low-density lipoprotein cholesterol and total cholesterol, and that KBs in CO can lower systolic blood pressure and improve insulin resistance [19], consuming large amounts of CO, like other saturated fats, can also be a precursor to cardiovascular diseases [22]. Numerous dietitians advise limiting, if not completely avoiding, CO intake due to its risks. CO is a complex combination that has been shown to modulate certain molecular processes related to AD, including redox status, energy metabolism, and the generation of Aβ [20].

## 4. Discussion

The results of this systematic review and meta-analysis suggest that CO has a positive effect on cognitive scores in AD compared with those of the control group. These results were consistent and statistically significant (*p* < 0.05). This review included a study by La Rubia Ort Je et al. [18], which concluded that patients with AD fared better cognitively after switching to a Mediterranean diet rich in CO. The study outcomes, indicating significant improvements in episodic and temporal orientation along with semantic memory, demonstrate the potential of an isocaloric Mediterranean diet enriched with CO to positively influence cognitive function among individuals with AD. The findings also revealed that while benefits were observed in both males and individuals with severe AD, females with mild-to-moderate AD most clearly benefited from the effects of CO. These findings show that dietary interventions based on CO may be an effective alternative to medication therapy in the management of AD [18]. Similarly, Yang et al. [21] provided additional evidence that CO improves cognitive function in patients with AD. After supplementation with extra virgin CO, the study participants’ cognitive test results, particularly those assessing orientation and language invention, showed statistically significant improvement. Notably, the benefits differed depending on the person’s sex, whether they had diabetes, and the severity of dementia. These findings highlight the necessity of customized approaches when examining the potential benefits of CO in patients with AD [21]. Moreover, De La Rubia Ort et al. [17] found that there was an improvement in cognitive abilities, particularly in memory, calculation, attention, orientation, and language development and repair following treatment with CO [17].

Medium-chain fatty acids (MCFAs), including lauric, caprylic, and capric acids, found in coconut oil (CO) are converted during metabolism into ketone bodies, such as β-hydroxybutyrate (β-HB) and acetoacetate (AcAc). These ketones provide neurons with an alternate energy source, which may improve cognitive function, especially in Alzheimer’s patients. Because of their neuroprotective qualities, medium-chain fatty acids (MCFAs) may lessen oxidative stress and inflammation. They may also modulate lipid metabolism, which may slow the conversion of amyloid precursor protein (APP) into amyloid beta (Aβ) [20]. Therefore, the potential advantages of CO for improving energy metabolism in the brain and influencing pathways linked to Aβ production and neuroinflammation are likely the source of CO’s cognitive benefits. These processes should be further investigated for potential therapeutic uses in the treatment of cognitive decline [20,22].

Juby et al. [22] have revealed that CO has a direct impact on the etiopathology of AD. This is linked to its antioxidant, anti-inflammatory, analgesic, anti-pyretic, anti-hepatotoxic [19], chemoprotective, and anti-hypercholesterolemic properties [20,22,23,24]. The restoration of brain antioxidant levels prevents additional neuronal damage and subsequent monoamine depletion [20]. According to Nafar et al. [25], CO treatment prevents the Aβ-induced decline in cell viability in cortical neurons in vitro and reduces the Aβ-induced elevation in ROS. CO has also been reported to increase the available energy to withstand cell stress, thereby aiding survival in the face of adversity [26]. Calsolaro and Edison have reported that neuroinflammation is a critical process in AD neurodegeneration [27]. Yeap et al. investigated the antioxidant benefits of VCO in vivo [28]. In addition, polyphenols present in CO have been reported to prevent lipid peroxidation in vitro [20], which may aid in the treatment of AD. In addition, VCO has been reported to have anti-stress and antidepressant properties owing to its high concentrations of MCFAs [29] and polyphenols [25]. However, Chan et al. (2017) found no significant improvements in cognitive scores after CO administration [16]. The volume of data in the included articles lends credence to the idea that various elements found in CO may have therapeutic advantages in AD management. The ability of oil to regulate oxidative stress, diminish neuroinflammation, improve mitochondrial function, and provide the brain with alternative energy sources is thought to be responsible for the neuroprotective properties of CO.

Variations in brain structure and function as well as hormonal effects can all have an impact on biological variances that can impact treatment response and cognitive processing. How one gender reacts to treatments may also depend on social and environmental factors, such as varying degrees of social support or participation in cognitive activities. There might be other reasons for the higher improvement in MMSE scores in women as opposed to males. Furthermore, results suggest that women could be more likely to ask for assistance and engage in health-related activities, which could result in better outcomes. These findings may potentially be influenced by individual differences in cognitive reserve and baseline cognitive performance.

Coconut oil (CO) is rich in medium-chain triglycerides (MCTs), which are metabolized into ketone bodies (KBs) that can serve as an alternative energy source for the brain, especially in cases of Alzheimer’s disease (AD) where glucose metabolism is impaired [22]. Fernando et al. [19] suggest that while MCTs in CO may not directly cross the blood–brain barrier, their metabolic byproducts, such as acetoacetate and β-hydroxybutyrate (β-HB), can support cognitive function by providing energy through alternative pathways. Additionally, CO has been observed to reduce the secretion of amyloid-β (Aβ) peptides, which are implicated in AD pathology, possibly by downregulating the transport protein ARF1. However, despite these potential benefits, the high saturated fat content of CO poses cardiovascular risks, leading to caution in its dietary use [20]. Ultimately, while CO may offer some neuroprotective effects, its overall impact on AD treatment is complex and varies among individuals.

While previous findings have suggested that females may experience more cognitive benefits from CO consumption, our results indicate otherwise. Except for in the verbal fluency test, male participants consistently scored higher and showed greater overall improvement after CO treatment compared to females [23,24,25]. This suggests that CO may have a more pronounced effect on male cognitive performance, warranting further investigation into potential gender-specific responses to CO interventions. Future studies should explore the underlying mechanisms that may account for these differences between men and women.

The potential therapeutic benefits of coconut oil are mostly dependent on its active ingredients, which include polyphenols, medium-chain triglycerides (MCTs), and medium-chain fatty acids (MCFAs). The antioxidant qualities of polyphenols may guard against oxidative stress, while MCTs and MCFAs can improve energy metabolism and give the brain another fuel source. Clarifying these elements and their physiological effects is crucial to understanding how coconut oil therapies might improve cognitive performance. We can evaluate the overall efficacy of coconut oil in therapeutic applications more accurately by establishing a connection between these active components and their particular effects.

Although these studies provide useful information, it is critical to recognize their limitations and the need for further research. The number of eligible studies meeting our inclusion criteria was limited, which restricted the scope of the analysis and the generalizability of the findings. Specifically, the small sample sizes, variations in study designs, and geographic distribution of the included studies could affect the overall results. Moreover, our search strategy did not include all other relevant databases, which may have resulted in the omission of ongoing or unpublished studies that could contribute to a more comprehensive analysis. Additionally, the potential for publication bias should be considered, as studies with negative results might not have been published. The studies included in this review showed discrepancies in origin, dietary patterns, and CO dosing. These differences make it challenging to generalize the findings to other populations. It is important to consider these variations when interpreting the results and applying them to broader contexts. Future studies should aim for more standardized methodologies to reduce heterogeneity and improve the reliability of the findings. In order to reduce the amount of variation in cognitive results, future research should standardize the amount and length of coconut oil treatments. Furthermore, measuring the plasma concentrations of medium-chain triglycerides (MCTs) and medium-chain fatty acids (MCFAs) before and after an intervention would shed light on their relationship with cognitive performance. Finally, maintaining uniform participant selection standards throughout research projects might contribute to the dependability and consistency of findings.

## 5. Conclusions

We conclude that, compared to the control group, CO improved cognitive scores in patients with AD, with the results being statistically significant (*p* < 0.05). The polyphenols, MCTs, and MCFAs present in CO are promising therapeutic agents for AD. The potential of CO as a non-pharmacological strategy for the management of AD has been demonstrated by cognitive improvements observed in patients with AD. With the deep dive that we undertook in this meta-review, it was found that CO can be used as a potential neuroprotective phytoconstituent; however, these studies can be dynamic. Significant gaps in research have been identified that need to be filled: various preclinical and human trials are taking place worldwide to ascertain the applicability of CO in humans; there is a lack of comparative findings on CO and its formulations against neuronal diseases, safety/toxicity data for CO, and clinical studies specifically exploring the pharmaceutical importance of CO for neuronal diseases.

## Figures and Tables

**Table 1:** Newcastle–Ottawa scale for non-randomized controlled trials.

Study	Selection				Comparability	Outcome			Quality
	Adequacy of Case Definition	Representativeness of the Cases	Selection of Controls	Definition of Controls	Comparability of Cases and Controls on the Basis of the Design or Analysis	Ascertainment of Exposure	Same Method of Ascertainment for Cases and Controls	Non-Response Rate	
Yang et al. [21]	✰	0	0	✰	✰	No Information	✰	✰	Good
Fernando et al. [19]	✰	0	0	✰	✰	No Information	✰	✰	Good
Juby et al. [22]	✰	0	0	✰	✰	No Information	✰	✰	Good
Guttmann et al. [20]	✰	0	0	✰	✰	No Information	✰	✰	Good

Note: ✰ show quality ratings.

**Table 2:** General information of included studies.

First Author, Year	Study Location	Study Design	Title	Objectives	Number of Study Participants	Intervention	Intervention Period	Control Group	Cognitive Assessments	Findings
Yang et al. [21]	Spain	Case-control	Coconut oil: alternative nonpharmacological treatment against AD	To assess coconut oil effects on Alzheimer’s dementia and factors including sex and diabetes	44 patients with AD out of a population of 458 patients evaluated	40 mL/d extra virgin coconut oil	21 days	Same feeding pattern as the case group, but without administration of oil	Mini-Examen Cognitivo (MEC)/MMSE	Improvement in cognitive abilities, with differences according to sex and disease severity
La Rubia Ortí Je et al. [18]	Spain	RCT	Improvement of main cognitive functions in patients with AD after treatment with coconut oil enriched Mediterranean diet: a pilot study	To search for improvements in primary cognitive function in individuals with AD after adopting a Mediterranean diet high in coconut oil	44 patients with AD	Coconut-oil-enriched Mediterranean diet		Standard low-fat diet	Seven-minute screen (7-MS)	Improvements in episodic, temporal orientation, and semantic memory in AD patients after the diet
De La Rubia Ortí et al. [17]	Spain	RCT	How does coconut oil affect cognitive performance in alzheimer patients?	To investigate coconut oil’s cognitive effects on individuals with AD in many cognitive domains	44 patients with AD	40 mL of extra virgin coconut oil daily	21 days	No oil	Cognitive test, MMSE	Improvement in cognitive abilities, with varying intensity depending on the cognitive area
Chan et al. [16]	Malaysia	RCT	Effect of cold pressed coconut oil on cognition and behavior among patients with AD–A pilot intervention study	To investigate the potential benefits of a dietary intervention for improving cognitive function in AD patients	40 individuals out of 99 screened	Coconut-oil-enriched Mediterranean diet	-	The control group received a placebo consisting of water plus coconut essence	MMSE and CDT	No significant difference in the mean scores in all the parameters assessed between the intervention and control group at baseline, 3 months, and 6 months
Guttmann et al. [20]	USA	Qualitative	Coconut Oil and its Constituents as a Treatment for Alzheimer’s Dementia	The properties of coconut oil as a therapeutic supplement for patients with Alzheimer’s disease	-	Coconut oil as a therapeutic supplement	-	-	CO has detrimental effects in addition to its potential to positively alter AD-related molecular pathways through KBs	Coconut oil and its constituents as a treatment for Alzheimer’s dementia (Coconut oil may have potential benefits in modifying Alzheimer’s disease-related pathways through ketone bodies; it also presents detrimental effects, which could include potential gastrointestinal discomfort, weight gain, and alterations in lipid profiles. Such side effects may overshadow the cognitive benefits in some patients.)
Juby et al. [22]	Canada	RCT, crossover study with an open label extension	Use of medium chain triglyceride (MCT) oil in subjects with Alzheimer’s disease: A randomized, double-blind, placebo-controlled, crossover study, with an open-label extension.	Use MCT oil supplementation to address the effect on cognition in subjects with mild to moderate AD	n = 20	-	-	Bulletproof Brain Octane (100% caprylic acid triglycerides, MCTs, from coconut oil)	Cognigram tests (1 and 2), MMSE, and MoCA	There was a statistically significant difference (*p* = 0.003) in the Cognigram 1 scores between the group of participants who had started with the placebo oil and those who had received 11 months of continuous MCT therapy
Fernando et al. [19]	Sri Lanka	Double-blind placebo-controlled trial	Effect of Virgin Coconut Oil Supplementation on Cognition of Individuals with Mild-to-Moderate Alzheimer’s Disease in Sri Lanka (VCO-AD Study): A Randomized Placebo-Controlled Trial	The influence of the apolipoprotein E (APOE) ɛ4 genotype on cognitive outcomes and the effect of VCO on cognition in AD patients	120 Sri Lankan individuals	30 mL/day of VCO orally and canola oil, for 24 weeks		The control group received a placebo consisting of VCO plus coconut essence	Cognition score was assessed using MMSE	Compared to canola oil, VCO did not enhance cognition in those with mild-to-moderate AD after a 24-week intervention. On the other hand, it raised the MMSE scores for APOE IV carriers

AD: Alzheimer’s disease; CDT: clock drawing test; MMSE: mini-mental state examination; RCT: randomized controlled trial.

**Table 3:** Mean MMSE scores (case vs. control) [18].

Experiment or Placebo	Pre-Test MMSE			Post-Test MMSE		
	Collective Mean	Male	Female	Collective Mean	Male	Female
Experiment	11.61	14.6	10.73	16.31	20	14.99
Placebo	11.42	11.67	11.38	11.56	12	11.49

MMSE, mini-mental state examination.

**Table 4:** Mean CDT scores, Benton’s test scores, verbal fluency test scores, and episodic memory test scores (case vs. control) (21).

Test	Group	Pre-Test Score	Post-Test Score	Statistical Significance
CDT Scores	Experiment	Male: 0.66		*p* < 0.05
		Female: 1.5	Female: 0.75	*p* < 0.05
	Placebo	Male: 0	Male: 0	-
		Female: 1	Female: 1	-
Benton’s Test Scores	Experiment	Male: 17.33	Male: 42	*p* < 0.05
		Female: 56.75	Female: 78	*p* < 0.01
	Placebo	Male: 7	Male: 12	*p* < 0.05
		Female: 77.33	Female: 50.33	*p* < 0.05
Episodic Memory Test Scores	Experiment	Male: 5.66	Male: 8	*p* < 0.01
		Female: 8.25	Female: 10	*p* < 0.05
	Placebo	Male: 4	Male: 10	*p* < 0.05
		Female: 7.5	Female: 6	-
Verbal Fluency Test Scores	Experiment	Male: 9.33	Male: 9.33	-
	Female: 7.5	Female: 9.5	*p* < 0.05	
	Placebo	Male: 5	Male: 7	-
		Female: 6.66	Female: 6.16	-

*p* = 0.03 can be noted instead of “*p* < 0.05” for more specificity.

## Data Availability

The data that support the findings of this study are available from Duaa Bafail upon reasonable request. Researchers interested in accessing the data can contact Duaa Bafail at dbafail@kau.edu.sa.

## Supplementary Materials

The following supporting information can be downloaded at: https://www.mdpi.com/article/10.3390/diseases12110272/s1, Table S1: Search strategy for MEDLINE (PubMed format).

## Abbreviations

CDT	Clock drawing test
AD	Alzheimer’s disease
MEC	Mini-Examen Cognoscitivo
MMSE	Mini-mental state examination
7MS	Seven-minute screen
SEM	Standard error of the mean
SMD	Standardized mean difference
CO	Coconut oil
VCO	Virgin coconut oil
MCT	Medium-chain triglyceride
MCFA	Medium-chain fatty acid
