# Supplementary material for: Impact of Coconut Oil and Its Bioactive Metabolites in Alzheimer’s Disease and Dementia: A Systematic Review and Meta-Analysis
Source: Diseases. 2024 Nov 1;12(11):272. doi: 10.3390/diseases12110272 (PMC11592914; doi:10.3390/diseases12110272)
Supplement: Supplementary file 1 [file diseases-12-00272-s001.zip › diseases-3199680-supplementary.pdf]

## SUPPLEMENTARY ITEMS:

**Table S1.** Search strategy for MEDLINE (PubMed format)

| Number | Search terms |
|---|---|
| #1 | Alzheimer [All Fields] |
| #2 | Alzheimer Disease [Mesh] |
| #3 | cognitive impair [All Fields] |
| #4 | neurocognitive disorder [All Fields] |
| #5 | memory loss [All Fields] |
| #6 | cognitive decline [All Fields] |
| #7 | Cognition Disorders [Mesh] |
| #8 | senile dementia [All Fields] |
| #9 | Senile Dementia [Mesh] |
| #10 | dementia [All Fields] |
| #11 | Dementia [Mesh] |
| #12 | #1 OR #2 OR #3 OR #4 OR #5 OR #6 OR #7 OR #8 OR #9 OR #10 OR #11 |
| #13 | MCT oil [All Fields] |
| #14 | medium chain triglycerides [All Fields] |
| #15 | lauric acid [All Fields] |
| #16 | caprylic acid [All Fields] |
| #17 | capric acid [All Fields] |
| #18 | coconut oil [All Fields] |
| #19 | Memory Disorders [Mesh] |
| #20 | Oils, Volatile [Mesh] |
| #21 | Medium-Chain Triglycerides [Mesh] |
| #22 | #13 OR #14 OR #15 OR #16 OR #17 OR #18 OR #19 OR #20 |
| #23 | #12 AND #22 |
